# Virtual Reality Hypnosis in the Electrophysiology Lab: When Human Treatments Are Better than Virtual Ones

**DOI:** 10.3390/jcm11133913

**Published:** 2022-07-05

**Authors:** Iklo Coulibaly, Laura Sofia Cardelli, Claire Duflos, Lionel Moulis, Bara Mandoorah, Jean Nicoleau, Leslie Placide, François Massin, Jean-Luc Pasquié, Mathieu Granier

**Affiliations:** 1 Cardiology Department, CHU Montpellier, 34090 Montpellier, France; iklocoulibaly@yahoo.fr (I.C.); dr_mandoorah@yahoo.com (B.M.); jean.nicoleau.34@gmail.com (J.N.); l-placide@chu-montpellier.fr (L.P.); f-massin@chu-montpellier.fr (F.M.); jl-pasquie@chu-montpellier.fr (J.-L.P.); 2 Cardiology Department, Ospedale Maggiore, 40133 Bologna, Italy; cardellilaura.med@gmail.com; 3 Clinical Research and Epidemiology Unit, CHU Montpellier, 34090 Montpellier, France; c-duflos@chu-montpellier.fr (C.D.); l-moulis@chu-montpellier.fr (L.M.); 4 National Institute of Health and Medical Research, Unit 1046, Montpellier University, 34090 Montpellier, France

**Keywords:** virtual reality hypnosis, virtual reality, hypnosis, electrophysiology, interventional cardiac electrophysiology

## Abstract

Aims: Virtual reality hypnosis (VRH) has been used successfully in various clinical settings to decrease anxiety and the sensation of pain. We aimed to investigate the feasibility and safety of VRH in patients undergoing electrophysiology and pacing procedures under conscious sedation. Methods: During a two-month period, VRH support was proposed and accepted by 25 patients undergoing electrophysiological procedures. Data were compared with a control group (*n* = 61) enrolled during the following three-month period. Both groups underwent the measurement of the duration of intervention, the consumption of analgesics and hypnotics, and their pain and comfort using a validated visual analogue scale (VAS 0−10). Results: The baseline characteristics were comparable in both groups, including age. There were no differences in procedure duration (46 (±29) vs. 56 (±32) min, *p* = 0.18) or in hypnotic/antalgic consumption (midazolam 1.95 (±1.44) vs. 2.00 (±1.22) mg, *p* = 0.83; sufentanyl 3.78 (±2.87) vs. 3.58 (±2.48) μg, *p* = 0.9) between the control and VRH groups. In a multivariate analysis, the use of VRH was independently associated with lower comfort during the procedure assessed by postoperative visual analogue scale (OR 15.00 [95% CI 4.77−47.16], *p* < 0.01). There was no influence of VRH use on pain or drug consumption. Conclusions: In our experience, compared with VRH, human care is preferable during procedures in electrophysiology lab to improve the comfort of the patient. VRH has no influence on pain or drug consumption.

## 1. Background

Virtual reality (VR) is a technology that allows a user to become immersed and to interact with an engrossing computer-generated environment. It was developed in the medical field in the early 2000s [1], mostly to reduce pain and anxiety in the perioperative period. Dedicated programs of virtual reality hypnosis (VRH) have been studied in burn-injured patients, trauma, urological treatment, and dental pain, but mostly in studies with a small number of patients [2,3,4,5,6].

The technique can be clinically used to draw the patient’s attention into the virtual world, distracting him/her and therefore leaving less attention available to process the nociceptive signals or pain [7]. As a non-invasive and supposedly non-addictive distractive analgesic technique, VRH has minimal side effects, making it a safe adjunct to pharmacological analgesics in the management of procedural pain and comfort.

In the field of interventional cardiology, potential interest has been demonstrated in VRH during transcatheter aortic valve replacement (TAVR) procedures [8]. Electrophysiological (EP) procedures and cardiac electronic device implantation can generate the same mindset in patients and are mostly performed under conscious sedation. One study on VRH in the cryoablation of atrial fibrillation (AF) has recently been published [9], but to the best of our knowledge, VRH has never been investigated for all types of routine procedures in the electrophysiology laboratory (EP lab).

We aimed to describe the feasibility of this technique in the setting of interventional electrophysiology and to examine the potential gains in comfort and pain reduction for the patient compared with conventional human care. In fact during all medical procedures, including electrophysiological ones, a direct relationship is created between the doctor, the nursing staff and the patient. Words and physical contact are often able to reassure the patient.

The main objective here was to assess the impact of the use of VRH on perioperative pain compared with usual human care in patients undergoing electrophysiology and cardiac stimulation under conscious sedation. Secondary objectives were to appraise the impact of VRH use on postoperative comfort and to analyse the factors influencing the analgesics and hypnotic doses received by the patients during the procedure.

## 2. Methods

Ethical approval for this study was obtained from the ethics committee of our institution (IRB 198711). All subjects were required to give written informed consent prior to participating in the study. The study was conducted at the EP lab of the cardiology department at Arnaud de Villeneuve Teaching Hospital in Montpellier, France. A retrospective, monocentric and comparative study was performed using a before–after design.

### 2.1. Population and Patient Selection

VR headsets were offered to all patients who were admitted to the EP lab during a two-month period (May–July 2020) and assessed for eligibility. 

Patients admitted to the EP lab were asked to use a VR headset (PICO Goblin2™, Singapore) with a dedicated VRH program (Cayceo™, Montpellier, France) associated with an audio headset: this allows the patient to view short video and hear audio sequences with a hypnotic and relaxing impact (see Supplementary Figure S1 and https://www.youtube.com/watch?v=bZFbvs5KCWU (accessed on 1 February 2020)). The virtual reality system produced the three-dimensional simulation of a natural environment; the surroundings could be traversed, creating a sense of immersion. This allowed the patient to have a complete and captivating sensory experience. During the procedure, doctors and nurses were advised to minimize vocal inferences and avoid contact with the patient to allow for greater concentration. 

We did not include for comparative analysis patients with deafness, blindness, or cognitive impairment, patients who refused the study, or patients who removed the VR mask during the procedure. The patients in the usual care group were enrolled during the next three months (August–October 2020). 

**Figure 1:**
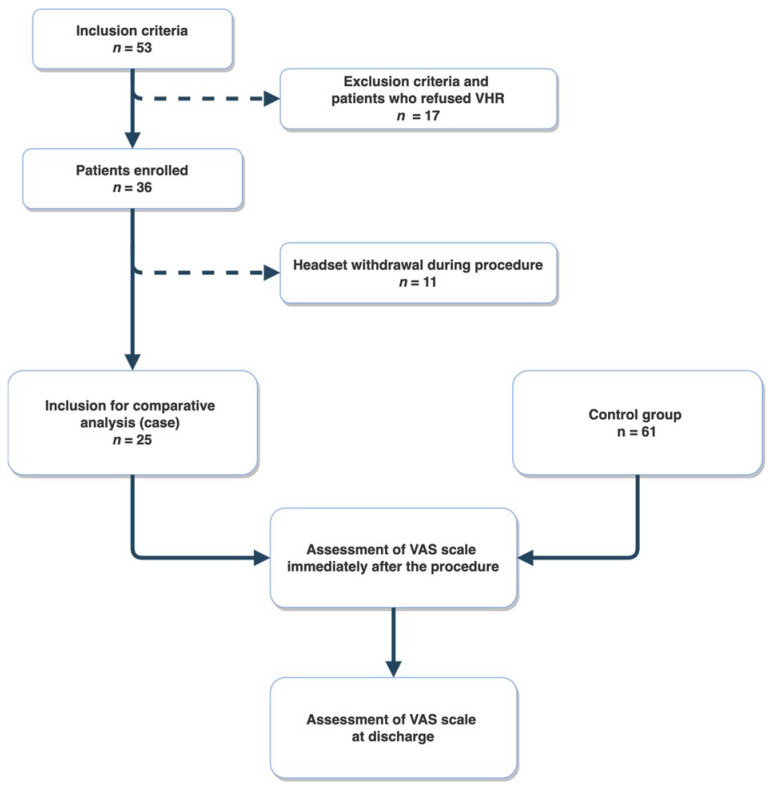
Flow chart of the study. VRH: virtual reality hypnosis. VAS: Visual Analogue Scale.

From the 53 patients assessed for eligibility, 17 patients refused the VR mask, and 11 removed the VR headset during the procedure. Finally, 25 patients (cases) were enrolled for a comparative analysis. The control group patients (*n* = 61) were enrolled during the following three-month period to allow for a 2:1 case-control comparison (Figure 1).

### 2.2. Study Procedure

In the EP lab, after a brief explanation by the nurse responsible for managing pain and comfort, the VR headset and mask were placed on the patient’s ears and eyes before the beginning of the procedure. 

Analgesic (sufentanyl) and hypnotic (midazolam) drugs were administered before the beginning of the procedure, and doses were increased according to the patient’s need during the procedure.

Immediately after the procedure (postoperative) and at hospital discharge, each patient was subjected to a visual analogue scale (VAS) on which the patients identified their levels of pain and comfort. The VAS is a numerical rating scale consisting of 10 gradations (1 for the most pain or the worst comfort, and 10 for the least pain and the best comfort) with an additional 10 sub-gradations for each point.

### 2.3. Statistical Analysis

The quantitative variables were presented as means (±standard deviation). The qualitative variables presented as total numbers and percentage.

The postoperative pain and comfort VAS scores did not follow a Gaussian distribution. Therefore, they were categorized in five quintiles, and we performed an ordinal logistic regression to assess the impacts of the use or not of VR, age, sex, type of procedure, duration of the procedure, dose of hypnotics, and dose of analgesics. No variables were selected to avoid the effect of overfitting on our estimates. The factors associated with the doses of hypnotics and analgesics during the procedure were assessed using multivariate linear regression with the stepwise selection of variables and an alpha threshold of 0.2.

## 3. Results

A total of 53 patients were screened to be enrolled in the VRH program, and 36 accepted wearing the VR headset. The reasons for refusal (*n* = 17, 32%) are listed in Table 1. More than half of the patients (*n* = 10) refused because of difficulties in understanding the VR setting. Throughout the procedure, patients were free to withdraw the headset for any reasons.

Among the 36 patients who accepted the VRH headset, 11 (30%) withdrew it during the procedure. The reasons for headset withdrawal are listed in Table 2. In most cases, the reason was discomfort or displacement. 

Finally, 25 patients in the VRH group and 61 patients in the control group were enrolled for comparative analysis. The characteristics of the population are shown in Table 3.

**Figure 2:**
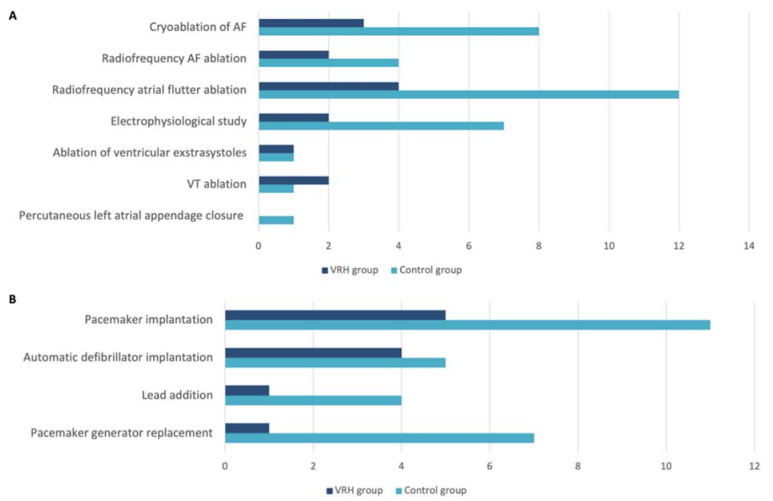
Specific electrophysiological procedure (**A**) and cardiac pacing procedure (**B**). AF: atrial fibrillation; VT: ventricular tachycardia; VRH: virtual reality hypnosis.

In the VRH group, 14 EP procedures and 11 cardiac pacing procedures (CP) were performed. In the control group, 34 EP procedures and 27 CP procedures were performed. The specific type of procedure per group is summarized in Figure 2A,B.

In patients undergoing VRH, the mean pain values measured with the VAS immediately after the procedure and at discharge were 3.7 (±2.2) and 2.1 (±2.9), respectively; the mean comfort values were 7.8 (1.8) and 7.9 (2.1), respectively. In the control group, the mean VAS pain values were 3.1 (±2.9) and 2.6 (±2.9) immediately after the procedure and at discharge, respectively; the mean comfort values were 9.5 (1.1) and 9.7 (0.7), respectively.

In the logistic regression model, the use of VRH did not have any influence on the postoperative pain VAS (OR 0.74, 95% CI 0.30–1.80, *p* = 0.51). Only the type of procedure and procedure duration were independently associated with the reduction of pain in the multivariate model (Table 4).

Furthermore, the use of VRH was independently associated with greater discomfort for the patient (OR 15.0, 95% CI 4.77–47.16, *p* < 0.01) (Table 5).

The results for pain and comfort remained consistent even at the time of discharge: VRH did not have any influence on pain VAS score (OR 1.45, 95% CI 0.54–3.20, *p* = 0.46) but was associated with a lower comfort VAS score (OR 9.81, 95% CI 3.01–32.03, *p* < 0.001) (see Supplementary Tables S1 and S2).

The use of VRH did not influence midazolam or sufentanyl consumption during the procedure (Table 6A,B). 

## 4. Discussion

VRH is widely used as an adjunctive therapy in various medical settings, and numerous reports have documented the potential analgesic benefits of immersive VR in medical settings ranging from cancer therapy to urological procedures and venous puncture in both children and adults [3,4,10,11,12]. The use of VRH is rare in interventional cardiology. 

VRH as an adjunctive tool in the alleviation of the patient’s pain and anxiety in a catheterization lab setting is seldomly used [8]. Our work analyses the potential use of VRH as an adjunctive therapy in patients undergoing interventional procedures in an electrophysiology laboratory. 

Roxburgh et al. recently published a similar study in a population undergoing the AF cryoablation [9], but our results are opposite of theirs, particularly in terms of procedural comfort. Their study recruited 48 patients, the duration of procedures was not reported, and the main endpoint was assessed 45 min after the end of the procedure in patients receiving narcotics, which could be a bias (for example memory bias).

Our study population represents the daily practice population in an EP lab. Consecutive and unselected patients were asked to participate in the study. However, patients without VRH tend to be older, even if non-significantly (*p* = 0.07). This highlights a tendency not to propose the VRH headset to older patients, generally deemed to be less receptive to such novel technologies. This probably is not a real limitation, as this approach has been reported as promising in patients admitted for TAVR [8].

Among the patients initially screened for enrolment, 17 (32%) were ultimately not included. This could be explained by the 68% feasibility as well as by physical limitations of the patients, although 6 (35.3%) refused without any physical reason (mainly due to fear or lack of explanation).

The VRH headset was removed by 11 (30.5%) patients during the procedure, for a percentage of acceptability of 69.5%. The main reason for removal was discomfort. We did not encounter typical “cybersickness” syndrome (defined as nausea, disorientation, blurred vision, and headaches) described in previous VR experiences [11]. However, some of these patients might have suffered from an incomplete cybersickness syndrome. Interestingly, among the 6 patients who removed the headset due to discomfort, 50% were undergoing AF cryoablation, which is usually longer than the other procedures. The statistical relationship between procedure duration and headset removal remains to be determined in a larger cohort.

In their work on VR in TAVR, Romano Bruno et al. [8] reported that 81.3% wore the VR headset until the completion of implantation and 37.5% until the end of the procedure. Our results appeared to be better in terms of headset acceptability. This difference could be due to a better acceptance of our item thanks to the association of a dedicated hypnosis program. 

Regarding the procedure duration, the study and control groups did not differ significantly. A 10 min difference was observed, which may reflect the time required to install and explain the VRH headset.

The use of VRH had no influence on either postoperative VAS score, or use of antalgic or hypnotic drugs. Because hypnosis is intended to help reduce pain perception, as demonstrated in various studies and conditions, this result might seem unexpected. In fact, in interventional and cardiac surgery, data on VRH effects are scarce. In a recent review of the literature, Rousseaux et al. [1] identified only eight studies addressing the effect of VRH on pain, only two of which were prospective randomized trials [5,13] with a significant number of healthy subjects. We did not find any large prospective randomized studies on VRH effects during interventional cardiology procedures, even if trials are ongoing in cardiac surgery [14]. Furthermore, many studies use VRH during the pre- or postoperative period, although rarely during the procedure. Regarding the effect on pain, Patterson et al. [5] investigated more specifically the different types of hypnoanalgesia rather than the effect of VRH itself on pain versus the absence of VRH. Regarding the effect on medication use, among three studies with a small number of patients, two report a reduction in opioid use [15,16], and one reports no modification [6].

In our results, there is no evidence that VRH has beneficial effects on medication use and pain reduction during EP procedures. However, the level of pain reported was low in both groups, as were the doses of medications. The VAS pain perception scores are in contradiction with those of Roxburgh et al., who found a significant difference between groups [9]: indeed, our absolute result for VAS score in the VRH group is quite close to Roxburgh et al.’s (3.74 in our study and 3.5 in their study, respectively), but the results in the two control groups are different (3.07 vs. 4.3, respectively).

Our main finding is that patients who accept VRH throughout the procedure report less comfort than the controls, and the results are confirmed by a second VAS measurement performed at discharge. With the same VAS scores in the VRH group compared with the control group, the results obtained immediately after the end of the procedure (7.94 vs. 9.67; *p* < 0.01) are opposite those of Roxburgh et al. obtained 45 min after the end of the procedure (7.5 vs. 6.8; *p* = 0.03). Even if we cannot fully exclude the impacts of different VR programs or study populations, these results advocate for the standardization of VRH and support the need for randomized studies in this field. 

Our finding could easily be explained as indicating the significant value of human care that can provide explanation, reassurance, and empathy as well as physical (eye and skin) contact, all by a professional nurse during the procedure. On the contrary, during the VRH program, the team present in the EP lab was required to avoid conversation and contact with the patient. Our results suggest that standard human care is more effective than the VRH program.

Future randomized studies with devices capable of allowing greater patient involvement are needed to definitively establish the role of VRH during electrophysiology procedures.

## 5. Limitations

This was a retrospective, monocentric, non-randomized trial. Substantial biases cannot be ruled out, and the refusal and withdrawal rates were approximately one third each. Furthermore, we cannot exclude that our study was underpowered to reach significant results.

Sufentanyl together with short-half-life benzodiazepines are routinely used to section patients in our electrophysiology laboratory, as well as in numerous interventional cardiology laboratories in France and Europe [17]. VRH has not been tested in the absence of sedation, and therefore, future studies will have to test its application in this setting.

## 6. Conclusions

In the setting of EP procedures in the EP lab under conscious sedation, we found that conventional human care is better than adjunctive VRH because it increases patient comfort with procedures. VRH has no influence on pain or drug consumption; procedure duration is non-significantly longer by a mean of ten minutes. One third of the patients refused the VRH, and one third had to remove the headset during the procedure. It is still unclear whether VRH can improve patient care. Future randomized trials will be needed to definitively prove the possible benefit of VRH.

## Figures and Tables

**Table 1:** Refusal reasons.

Patients	Non-Inclusion Reasons
N°1	Obnubilation
N°3	Hypoacousy
N°7	Hypoacousy
N°8	Refusal
N°11	Refusal
N°12	Hypoacousy
N°13	Lack of comprehension
N°14	Refusal
N°25	Lack of comprehension
N°27	Hypoacousy
N°29	Non-compliance
N°33	Language barrier
N°34	Refusal
N°36	Language barrier
N°39	Refusal
N°41	Deafness
N°49	Refusal

**Table 2:** Headset removal reasons.

Patients	Reasons
N°5	Discomfort
N°15	Discomfort
N°17	Discomfort
N°18	Calibration issue
N°20	Calibration issue
N°22	Not available
N°23	Headset displacement
N°32	Headset displacement
N°35	Discomfort
N°45	Discomfort
N°53	Discomfort

**Table 3:** Baseline characteristics of the population and the group comparison. VRH: virtual reality hypnosis; VAS: visual analogue scale. The VAS consisted of 10 score gradations (1 for the most pain or the worst comfort and 10 for the least pain and the best comfort) with an additional 10 sub-gradations for each point.

	Total Population N = 86	With VRH N = 25	Without VRH N = 61	*p*
Female sex, *n* (%)	20 (23)	6 (24)	14 (22)	0.92
Stimulation procedure, *n* (%)	39 (45)	11 (44)	28 (45)	0.87
Age (years)	66 (±16)	61 (±17)	69 (±14)	0.07
Procedure duration (min)	49 (±30)	56 (±32)	46 (±29)	0.18
Midazolam dose (mg)	1.96 (±1.37)	2.00 (±1.22)	1.95 (±1.44)	0.83
Sufentanyl dose (µg)	3.72 (±2.75)	3.58 (±2.48)	3.78 (±2.87)	0.9
Pain VAS post-procedure (0–10 score)	3.26 (±2.72)	3.74 (±2.21)	3.07 (±2.89)	0.25
Comfort VAS post-procedure (0–10 score)	9.01 (±1.54)	7.83 (±1.79)	9.47 (±1.15)	<0.01
Pain VAS at discharge (0–10 score)	2.45 (±2.87)	2.11 (±2.90)	2.57 (±2.88)	0.52
Comfort VAS at discharge (0–10 score)	9.23 (±1.42)	7.94 (±2.09)	9.67 (±0.70)	<0.01

**Table 4:** Univariate and multivariate analysis results for VRH use regarding the perception of procedural pain assessed with a postoperative visual analogue scale (VAS). VRH: virtual reality hypnosis; EP procedure: electrophysiological procedure; OR: odds ratio; CI: confidence interval.

	Univariate		Multivariate	
Variable	OR (95% CI)	*p*	OR (95% CI)	*p*
VRH group	0.58 (0.25–1.35)	0.21	0.74 (0.30–1.80)	0.51
Age (years)	1.01 (0.99–1.04)	0.28	1.00 (0.97–1.03)	0.84
Female sex	0.66 (0.26–1.63)	0.36	0.62 (0.24–1.66)	0.35
EP Procedure	0.41 (0.19–0.90)	0.03	0.32 (0.14–0.77)	0.01
Duration (min)	0.98 (0.97–1.00)	0.01	0.98 (0.97–1.00)	0.03
Midazolam dose (mg)	0.75 (0.56–1.00)	0.05	1.00 (0.67–1.50)	1.00
Sufentanyl dose (µg)	0.83 (0.72–0.96)	0.01	0.90 (0.75–1.08)	0.27

**Table 5:** Univariate and multivariate analysis results for VRH use regarding the comfort of the procedure assessed with a postoperative visual analogue scale (VAS). VRH: virtual reality hypnosis; EP procedure: electrophysiological procedure; OR: odds ratio; CI: confidence interval.

	Univariate		Multivariate	
Variable	OR (95% CI)	*p*	OR (95% CI)	*p*
VRH group	13.87 (4.78–40.28)	<0.01	15.00 (4.77–47.16)	<0.01
Age (years)	0.99 (0.97–1.02)	0.68	1.02 (0.98–1.06)	0.31
Female sex	0.87 (0.32–2.41)	0.79	0.71 (0.22–2.30)	0.56
EP Procedure	0.90 (0.39–2.08)	0.81	0.98 (0.36–2.67)	0.97
Duration (min)	1.01 (1.00–1.03)	0.13	1.01 (0.99–1.02)	0.59
Midazolam dose (mg)	1.11 (0.81–1.50)	0.52	1.23 (0.77–1.94)	0.39
Sufentanyl dose (µg)	1.01 (0.87–1.18)	0.88	0.97 (0.78−1.20)	0.76

**Table 6:** Hypnotic and antalgic use. A: Influencing factors of Midazolam use. B: Influencing factors of sufentanyl use. VRH: virtual reality hypnosis; EP procedure: electrophysiological procedure; CI: confidence interval.

**(A)**						
	**Univariate**			**Multivariate**		
**Variable**	**Beta**	**95% CI**	***p***	**Beta**	**95% CI**	***p***
VRH group	0.05	−0.60; 0.71	0.88			
Age (years)	−0.03	−0.05; −0.02	<0.01	−0.03	−0.04; −0.01	<0.01
Female sex	−0.18	−0.88; 0.53	0.61			
EP Procedure	0.60	0.02; 1.20	0.04			
Duration (min)	0.01	0.00; 0.02	0.02	0.01	0; 0.01	0.19
Sufentanyl dose (µg)	0.30	0.21; 0.39	<0.01	0.25	0.16; 0.34	<0.01
**(B)**						
	**Univariate**			**Multivariate**		
**Variable**	**Beta**	**95% CI**	***p***	**Beta**	**95% CI**	***p***
VRH group	−0.20	−1.51; 1.10	0.76			
Age (years)	−0.02	−0.06; 0.01	0.19			
Female sex	0.10	−1.31; 1.50	0.89			
EP Procedure	0.91	−0.26; 2.09	0.12			
Duration (min)	0.03	0.01; 0.05	<0.01	0.02	0.00; 0.03	0.05
Midazolam dose (mg)	1.20	0.85; 1.55	<0.01	1.11	0.71; 1.53	<0.01

## Data Availability

The data presented in this study are available on request from the corresponding author. The data are not publicly available due to privacy reason.

## Supplementary Materials

The following supporting information can be downloaded at: https://www.mdpi.com/article/10.3390/jcm11133913/s1, Table S1: Univariate and multivariate analysis of VRH use regarding the pain of the procedure at discharge assessed by post-operative VAS. Table S2: Univariate and multivariate analysis of VRH use regarding the comfort of the procedure at discharge assessed by post-operative VAS. Figure S1: Patient with virtual reality equipment during electrophysiological procedure.

## Abbreviations

VR	Virtual reality
VRH	Virtual reality hypnosis
EP	Electrophysiological
EP lab	Electrophysiology laboratory
VAS	Visual analogue scale
CP	Cardiac pacing
TAVR	Transcatheter aortic valve replacement
AF	Atrial fibrillation

## References

[1] Rousseaux F., Bicego A., Ledoux D., Massion P., Nyssen A.S., Faymonville M.E., Laureys S., Vanhaudenhuyse A. (2020). Hypnosis Associated with 3D Immersive Virtual Reality Technology in the Management of Pain: A Review of the Literature. J. Pain Res..

[2] Das D.A., Grimmer K.A., Sparnon A.L., McRae S.E., Thomas B.H. (2005). The efficacy of playing a virtual reality game in modulating pain for children with acute burn injuries: A randomized controlled trial [ISRCTN87413556]. BMC Pediatr..

[3] Hoffman H.G., Garcia-Palacios A., Patterson D.R., Jensen M., Furness T., Ammons W.F. (2001). The effectiveness of virtual reality for dental pain control: A case study. Cyberpsychology Behav. Impact Internet Multimed Virtual Real Behav. Soc..

[4] Wright J.L., Hoffman H.G., Sweet R.M. (2005). Virtual reality as an adjunctive pain control during transurethral microwave thermotherapy. Urology.

[5] Patterson D.R., Hoffman H.G., Palacios A.G., Jensen M.J. (2006). Analgesic effects of posthypnotic suggestions and virtual reality distraction on thermal pain. J. Abnorm. Psychol..

[6] Teeley A.M., Soltani M., Wiechman S.A., Jensen M.P., Sharar S.R., Patterson D.R. (2012). Virtual reality hypnosis pain control in the treatment of multiple fractures: A case series. Am. J. Clin Hypn..

[7] Hoffman H.G., Seibel E.J., Richards T.L., Furness T.A., Patterson D.R., Sharar S.R. (2006). Virtual Reality Helmet Display Quality Influences the Magnitude of Virtual Reality Analgesia. J. Pain..

[8] Bruno R.R., Lin Y., Wolff G., Polzin A., Veulemans V., Klein K., Westenfeld R., Zeus T., Kelm M., Jung C. (2020). Virtual reality-assisted conscious sedation during transcatheter aortic valve implantation: A randomised pilot study. EuroIntervention.

[9] Roxburgh T., Li A., Guenancia C., Pernollet P., Bouleti C., Alos B., Gras M., Kerforne T., Frasca D., Le Gal F. (2021). Virtual Reality for Sedation During Atrial Fibrillation Ablation in Clinical Practice: Observational Study. J. Med. Internet Res..

[10] Gershon J., Zimand E., Lemos R., Rothbaum B.O., Hodges L. (2003). Use of Virtual Reality as a Distractor for Painful Procedures in a Patient with Pediatric Cancer: A Case Study. CyberPsychol. Behav..

[11] Gershon J., Zimand E., Pickering M., Rothbaum B.O., Hodges L. (2004). A Pilot and Feasibility Study of Virtual Reality as a Distraction for Children With Cancer. J. Am. Acad. Child. Adolesc. Psychiatry.

[12] Windich-Biermeier A., Sjoberg I., Dale J.C., Eshelman D., Guzzetta C.E. (2007). Effects of distraction on pain, fear, and distress during venous port access and venipuncture in children and adolescents with cancer. J. Pediatr. Oncol. Nurs. J. Assoc. Pediatr. Oncol. Nurses.

[13] Enea V., Dafinoiu I., Opriş D., David D. (2014). Effects of hypnotic analgesia and virtual reality on the reduction of experimental pain among high and low hypnotizables. Int. J. Clin. Exp. Hypn..

[14] Rousseaux F., Faymonville M.E., Nyssen A.S., Dardenne N., Ledoux D., Massion P.B., Vanhaudenhuyse A. (2020). Can hypnosis and virtual reality reduce anxiety, pain and fatigue among patients who undergo cardiac surgery: A randomised controlled trial. Trials.

[15] Patterson D.R., Wiechman S.A., Jensen M., Sharar S.R. (2006). Hypnosis delivered through immersive virtual reality for burn pain: A clinical case series. Int. J. Clin. Exp. Hypn..

[16] Patterson D.R., Tininenko J.R., Schmidt A.E., Sharar S.R. (2004). Virtual reality hypnosis: A case report. Int. J. Clin. Exp. Hypn..

[17] Hamid A. (2014). Anesthesia for cardiac catheterization procedures. Heart Lung Vessel.

