# Supplementary material for: Virtual Reality Hypnosis in the Electrophysiology Lab: When Human Treatments Are Better than Virtual Ones
Source: J Clin Med. 2022 Jul 5;11(13):3913. doi: 10.3390/jcm11133913 (PMC9267480; doi:10.3390/jcm11133913)
Supplement: Supplementary file 1 [file jcm-11-03913-s001.zip › jcm-1739370-supplementary.pdf]

Supplementary material

Presentation of the dedicated HVR programme: https://www.youtube.com/watch?v=bZFbvs5KCWU

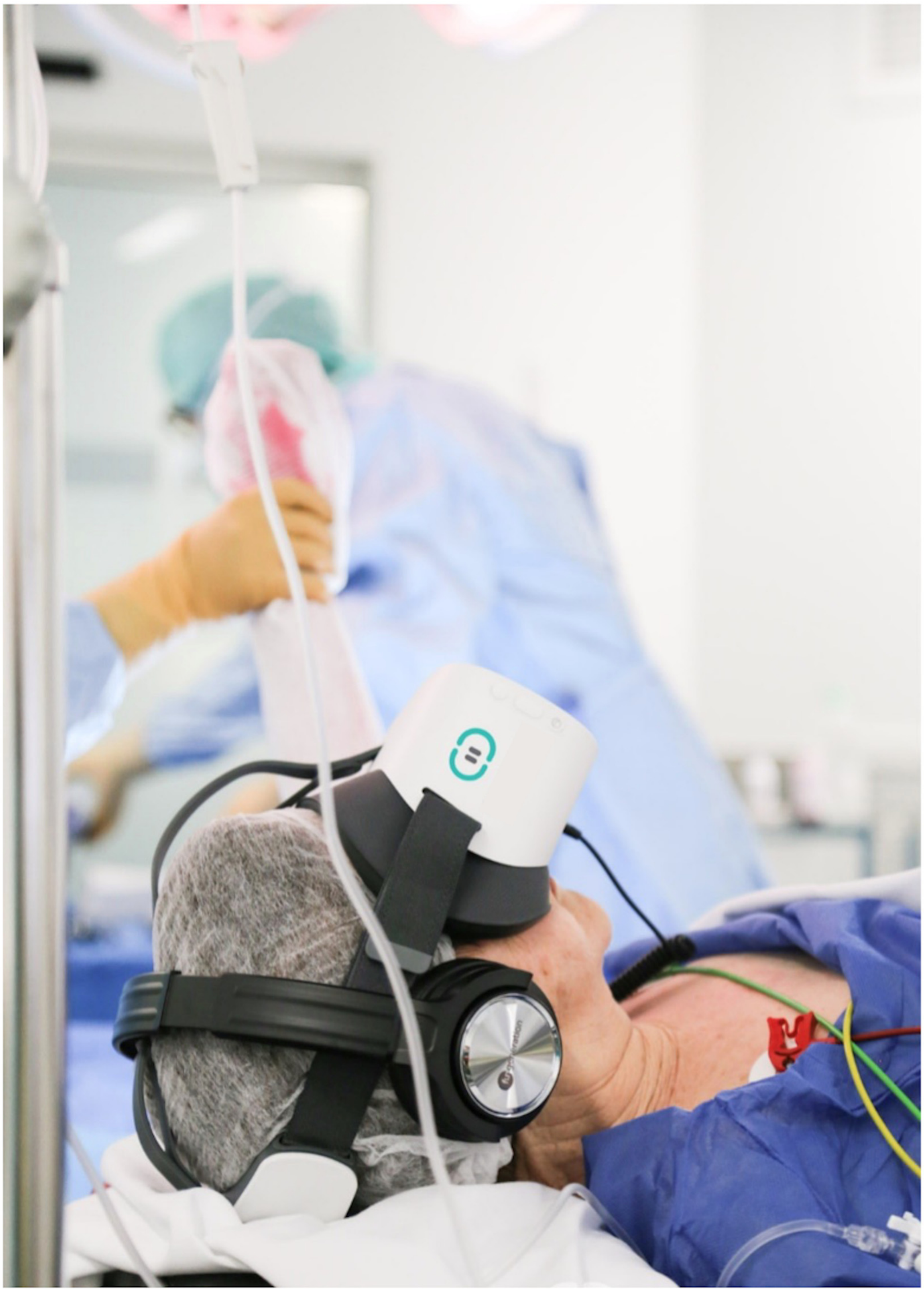

Figure S1 - Patient with virtual reality equipment during electrophysiological procedure.

Table S1: Univariate and multivariate analysis of VRH use regarding the pain of the procedure at discharge assessed by post-operative visual analogue scale (VAS). VRH: virtual reality hypnosis; EP procedure: electrophysiological procedure; CI: confidence interval.

	Univariate		Multivariate	
Variable	OR (95% CI)	*p*	OR (95% CI)	*p*
VRH group	1.28 (0.51-3.22)	0.60	1.45 (0.54-3.20)	0.46
Age (years)	1.00 (0.97-1.03)	0.91	0.99 (0.95-1.02)	0.46
Female sex	0.46 (0.18-1.19)	0.11	0.42 (0.15-1.19)	0.10
EP Procedure	1.21 (0.54-2.69)	0.64	0.98 (0.36-2.67)	0.97
Duration (min)	0.99 (0.97-1.00)	0.07	0.99 (0.97-1.00)	0.14
Midazolam dose (mg)	0.85 (0.63-1.14)	0.28	1.23 (0.77-1.94)	0.39
Sufentanyl dose (µg)	0.91 (0.78-1.05)	0.19	0.95 (0.78-1.16)	0.61

Table S2: Univariate and multivariate analysis of VRH use regarding the comfort of the procedure at discharge assessed by post-operative visual analogue scale (VAS). VRH: virtual reality hypnosis; EP procedure: electrophysiological procedure; CI: confidence interval.

	Univariate		Multivariate	
Variable	OR (95% CI)	*p*	OR (95% CI)	*p*
VRH group	8.79 (3.06-25.23)	<0.0001	9.81 (3.01-32.03)	0.0002
Age (years)	0.99 (0.96-1.02)	0.64	1.01 (0.97-1.06)	0.54
Female sex	0.91 (0.30-2.74)	0.86	0.71 (0.19-2.75)	0.54
EP Procedure	0.67 (0.27-1.66)	0.39	0.57 (0.19-1.76)	0.33
Duration (min)	1.01 (0.99-1.02)	0.31	1.00 (0.98-1.02)	0.67
Midazolam dose (mg)	1.2 (0.86-1.66)	0.28	1.48 (0.88-2.48)	0.14
Sufentanyl dose (µg)	1.02 (0.85-1.18)	0.98	0.97 (0.76-1.23)	0.78
